# The Luminescence of Laser-Produced Carbon Nanodots: The Effect of Aggregation in PEI Solution

**DOI:** 10.3390/ma17071573

**Published:** 2024-03-29

**Authors:** Agata Kaczmarek, Agnieszka Wisniewska, Tomasz Mościcki, Jacek Hoffman

**Affiliations:** 1 Institute of Fundamental Technological Research, Polish Academy of Sciences, Pawinskiego 5B, 02-106 Warsaw, Poland; tmosc@ippt.pan.pl; 2 Institute of Physical Chemistry, Polish Academy of Sciences, Kasprzaka 44/52, 01-224 Warsaw, Poland; awisniewska@ichf.edu.pl

**Keywords:** aggregation-induced emission, carbon nanodots, polyethyleneimine, intrinsic fluorescence, adsorption kinetic study

## Abstract

Carbon nanodots (CNDs) produced in pure water by the ablation of graphite with a nanosecond laser pulse exhibit weak photoluminescence. A small addition of polyethyleneimine (PEI) to the aqueous suspension of CNDs causes a significant increase in emissions. This paper presents experimental and theoretical studies of the emission properties of CND/PEI systems. The obtained CNDs responded to even trace amounts of PEI in solution (~0.014% *v*/*v*), resulting in a significant increase in the initial weak blue emission of CNDs and PEI taken separately. Morphology and size measurements showed that particle aggregation occurred in the presence of the polymer. A decrease in the calculated Stokes shift values was observed with increasing PEI content in the solution. This indicates a reduction in the number of non-radiative transitions, which explains the increase in the emission intensity of the CND/PEI systems. These results therefore confirmed that the increase in the emission of CND/PEI systems is caused by particle aggregation. Kinetic studies proved that the process is controlled mainly by diffusion, the initial stage of which has a dominant influence on determining the optical properties of the system.

## 1. Introduction

Recently, research attention has been focused on the excellent optical properties of carbon nanodots (CNDs). The application potential of this material is based on the low toxicity of carbon and its biocompatibility. Moreover, since their discovery, carbon nanodots have been believed to exhibit very high emission quantum yield comparable to conventional luminescent quantum dots. Therefore, for many years, research emphasis has been placed on the development of new synthesis routes and novel fascinating applications in imaging, sensing, and therapeutics [1].

However, the relationship between the structure and optical properties of CNDs is still unclear [1,2,3]. Furthermore, a consensus on basic and versatile emission mechanisms is still lacking. Since multiple parameters can influence the optical properties of CNDs, such as the choice of synthesis approach, the selection of the precursor, the presence of various reagents, and post-processing, it is postulated that carbon nanodots represent a more complex system than anticipated. Therefore, the concept of a unified emission mechanism should be reconsidered [1].

In general, there are three emission mechanisms considered [2,3,4]:

- *Core emission*, determined by its degree of crystallinity and attributed to the π–π* transitions of the C=C bonds;
- *Surface states*, related to the presence of functional groups connected to the carbon core;
- *Molecular states*, where emission originates from free or bounded fluorescent molecules and is dramatically influenced by the nature (properties) of these luminophores.

Moreover, these ‘conventional’ mechanisms can be complemented with the following process:

- *Aggregation-induced emission* (AIE) resulting from the clustering of carbon nanodots. As a consequence, vibration and rotation motion are reduced, which results in an increase in the number of radiative transitions [5,6].

An example of the photoluminescence enhancement of a colloid containing carbon nanostructures due to aggregation is presented in a previous study [7]. In this work, a significant increase in the fluorescence of s-GQDs (single-layered graphene quantum dots) in the presence of Al^3+^ was found. The explanation for this phenomenon is that aggregation, by limiting the number of degrees of freedom, also reduces the available number of non-radiative transitions. Therefore, an increase in fluorescence and QY occurs [7].

Another example illustrating ambiguities of CNDs’ optical properties is the (in)dependency of the emitted light on the excitation wavelength. There are a number of works whose authors claim that carbon nanodots show tunable emission [1,8,9,10,11]. According to [12,13], the excitation wavelength dependence may result from the inhomogeneity of the structure, i.e., particles of different sizes may be present in the solution, and various functional groups can be found on the surface of the dots. Hence, each component of the dot will be optically activated by different wavelengths. However, there are publications whose authors postulate the independence of the emission from the excitation wavelength [14].

Another issue is the ability to control the optical properties of CNDs through various functional groups attached to their surface. Hence, the solvent-dependent properties of carbon nanodots are debatable. In the literature, one can find papers in which the influence of various surface groups has been discussed [9,10,15,16]. However, for several years, there has been increasing evidence that luminescence predominately occurs due to fluorescent molecules (fluorophores) [17], which are not bonded to carbon nanodots, as they emerge as side products of chemical synthesis. In order to support the filtration and purification of sole nanomaterials, the post-treatment of the obtained synthesis products has to be incorporated. However, these redundant molecules are difficult to dispose of, and hence, the final filtration product is never composed of carbon nanodots only. Nevertheless, purified nanomaterials had much lower emission yields (in order of magnitude) [17]. Even though pulsed laser ablation in liquids (PLALs) is perceived as an alternative to chemical syntheses, owing to limiting the amount of the reagent used, the presence of fluorophores cannot be excluded. This is due to the interactions between the laser beam and the surrounding liquid during the ablation process [10,18]. Hence, the source of the luminescence of the obtained CND complexes remains disputable.

In [19], it was found that, in the examined case, the strong fluorescence comes from fluorophores. Moreover, the authors state that the source of strong fluorescence is almost certainly fluorophores in other cases as well; therefore, “For other carbon dots with high fluorescence QY (Quantum Yield), their compositions and effective fluorescence components should be carefully examined before attributing the high QY to carbonaceous nanoparticles” [19].

On the other hand, there are authors stating that the functional group can affect the intensity of the emitted light while not significantly affecting the emission wavelength [20]. In the case of CNDs formed from cross-linked polymers (i.e., polyethyleneimine) [21], the change in emission (compared to a pure polymer solution as a reference) was due to the rigidifying of the polymer structure by CNDs and the increase in the number of radiative transitions. It should be noted, however, that the intensity of the emitted light increased, whereas the emission wavelength remained the same. As can be seen, contrary to common belief, the enhancement of the luminescence of the final product was due to the presence of CNDs. Also, this indicates that when considering the emission mechanisms of CND/reactant systems, the luminescence of the reactant itself should be taken into consideration.

Polyethyleneimine (PEI) has been extensively studied as one of the materials used to modify the properties of CNDs [10,21]. In drug and gene delivery systems, PEI is considered the golden standard for non-viral nucleic acid delivery, providing efficient binding to the cell surface, the endosomal release of the cargo, and translocation to the nucleus [22]. However, unmodified PEI suffers from cytotoxicity and lack of biodegradability [23]. To improve biocompatibility and enhance its unique features, PEI has been modified by carbon nanodots [24,25].

Research conducted in recent years on polyethyleneimine (PEI) and other polymers shows that, although they are not traditional luminophores (i.e., they do not contain a system of conjugated π-type bonds), they exhibit the so-called intrinsic fluorescence [26,27]. This phenomenon occurs in the case of polymers that contain ‘electron-rich’ heteroatoms in their structure, i.e., nitrogen; oxygen; sulfur; phosphorus; and/or unsaturated C=C, C≡N, and C=O bonds. These bonds can emit independently or as amides, imides, and esters. It should be noted that these compounds do not emit light under ‘normal’ conditions (i.e., autonomously). In the polymer, however, they form a larger cluster—a macromolecule incorporated into a polymer chain—and in this form, they start to undergo luminescence [26,28]. It is assumed that the aggregation and agglomeration of unconventional chromophores play a key role in the intrinsic emission process. The formation of larger clusters results in the overlapping electron clouds of C=O and –OH groups within the aggregates. The aggregation of carbonyl groups also reduces vibrational motions and reduces the number of non-radiative transitions. It has also been noted that the emission depends on the amount and type of chromophores attached to the polymer chain. The greater the variety of subgroups, the more clusters there are, and the greater their attraction, which leads to the strengthening/rigidifying of the polymer chain and, consequently, higher emission intensities [26].

Here, a study on the emission mechanism of CND/PEI systems is presented. A theoretical investigation of adsorption kinetics was carried out to elucidate the relation between the aggregation and temporal evolution of the emission of CND/PEI systems. Pristine aqueous carbon nanodots were obtained by laser ablation in water and mixed afterward with a PEI aqueous solution. The division of the process is supposed to address the issue of the excessive amounts of polymer (and the formation of fluorophores) used in previous research. Hence, the aqueous suspension of CNDs was used as a batch probe for further PEI functionalization. Three low polymer concentrations were utilized (0.014–1.67% *v*/*v*), and the effect of the polymer concentration on the change in morphology, shape, size, emission intensity, and absorbance was explored.

## 2. Experimental Procedures

In order to obtain CND/PEI systems, the synthesis route was divided into two stages, with the first step devoted to the production of pristine aqueous CNDs and the second one to the functionalization of as-obtained CND suspensions with the PEI aqueous solution.

### 2.1. Reagents and Materials

For ablation, a graphite target (99.997% pure, Goodfellow, UK) was used. For the experiments, the following reagents were used: ultrapure (deionized, DI) water obtained from Hydrolab HLP 10 (Gdańsk, Poland) apparatus and liquid branched polyethyleneimine (PEI, average Mw ~800, MERCK) in the following concentrations (*v*/*v*): 0.0014%, 0.014%, 0.25%, 0.33%, 1.67%, 2%, and 10%. For simplicity, these PEI solutions will be denoted in this paper as follows: PEI0.0014, PEI0.014, PEI0.25, PEI0.33, PEI1.67, PEI2, and PEI10, respectively.

### 2.2. Synthesis of Carbon Nanodots in Water

Aqueous carbon nanodots solutions were obtained via pulsed laser ablation in water using the procedure described in [29].

In order to dispose of any emerging larger particles, the obtained CND suspensions were centrifuged in a Micro CD2012 Centrifuge (Phoenix instrument, Garbsen, Germany). Centrifugation was performed for 60 min with 14,000 rpm. The resulting supernatants were used for further functionalization and analysis and will be referred to in this paper as CNDs(aq).

### 2.3. Modification (Functionalization) of CNDs(aq)

PEI0.25, PEI2, and PEI10 were mixed with CNDs(aq) to obtain the following concentrations of PEI in the mixture: 0.014%, 0.33%, and 1.67%, respectively. No further processing of samples was carried out. The concentration of carbon nanodots in all CND/PEI systems was kept constant (0.5mg/mL). This value was obtained based on the ablation yield calculations presented in [29]. Table 1 summarizes sample names, the procedure of mixing and respective concentration of PEI in mixture.

### 2.4. Characterization Methods

The hydrodynamic radius of the nanodots was determined using dynamic light scattering (DLS) with a Brookhaven 90 Plus Particle Size Analyzer (Brookhaven Instruments Corp., USA). All measurements were performed at 25 °C with a laser wavelength of 657 nm and 90° scattering angle. The size of pristine (aqueous) CNDs was measured directly after centrifugation, whereas the size of PEI-modified particles was measured 7 and 14 days after functionalization to gain information on the colloidal stability of samples.

The morphology of the samples was investigated using a scanning electron microscope (JSM-6390LV, JEOL GmbH, Freising, Germany)) and a transmission electron microscope (JEM-1011, JEOL GmbH, Freising, Germany)) with an FEI Titan instrument, operating at 300 kV, equipped with a field-emission gun (FEG) and a spherical aberration corrector system (Cs-corrector) of the objective lens. Before SEM and TEM analyses, the samples were drop-casted onto a carbon-coated 300-mesh copper grid and left to evaporate at room temperature for 24 h.

Absorption, emission, and excitation spectra were recorded with a spectrophotometer (Thermo Scientific Multiscan GO, USA) and a spectrofluorimeter (FS 5, Edinburgh Instruments, Edinburgh, UK), respectively. Absorbance was measured in the wavelength range of 200–800 nm, emission spectra were collected for an excitation wavelength of 350 nm, and excitation scans were acquired for the following emission wavelengths: 410, 430, 450, 470, and 490 nm. All optical spectra were recorded using quartz cuvettes (10 mm path length). Optical spectra were gathered immediately and within 18 days after synthesis/functionalization in order to provide insight into the temporal evolution of the optical properties of the systems.

The absorbance spectra of CNDs(aq) were corrected by subtracting the contribution of water. The absorbance spectra of PEI-modified samples were corrected by subtracting contributions of the CNDs(aq) and the reagent (i.e., the PEI solution with a concentration corresponding to the one in the CND/PEI mixture).

## 3. Results and Discussion

### 3.1. Characterization of Pristine Aqueous CNDs

**Figure 1:**
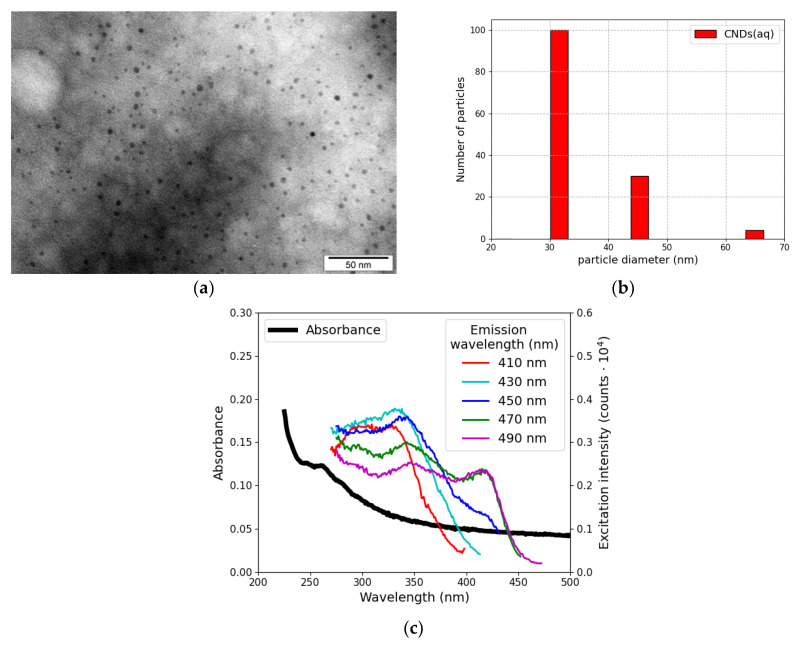
Characterization of CNDs(aq): morphology (TEM image) (**a**), DLS size distribution (**b**), UV–Vis absorption spectrum, and excitation spectra recorded for progressively longer emission wavelengths with 20 nm increments (**c**).

The obtained aqueous CNDs had spherical shapes and sizes of 3–5 nm, as shown in the TEM image presented in Figure 1a. Moreover, unmodified particles did not form agglomerates and were uniformly distributed throughout the sample. These results are consistent with [27].

However, the size distribution of CNDs (Figure 1b) obtained by dynamic light scattering did not indicate monodispersion, and the average size of nanodots was around 36 nm. The difference between TEM and DLS measurements can be explained by the absence or presence of the hydration layer on the surface of CNDs, respectively. DLS allows us to acquire the hydrodynamic radius of the particle, while TEM enables the estimation of the projected area diameter. An additional key difference between these techniques is that TEM is a number-based observation, whereas DLS is usually an intensity-based one. Therefore, the direct intensity size distribution may inherently be weighted to larger sizes than the number distribution, due to the fact that the scattering intensity is proportional to the sixth power of the particle radius [30].

The as-prepared CNDs(aq) had a UV absorption band located at 260 nm (Figure 1c). This peak is usually attributed to the $\pi -\pi^{*}$ transition of nanocarbon [5]. Additionally, carbon nanodots displayed excitation-wavelength-dependent emission, although in the recorded emission range, i.e., 410–490 nm, the excitation wavelength changed only slightly (from 320 to 350 nm). Also, emission intensities were low.

### 3.2. Characterization of CND/PEI Systems

**Figure 2:**
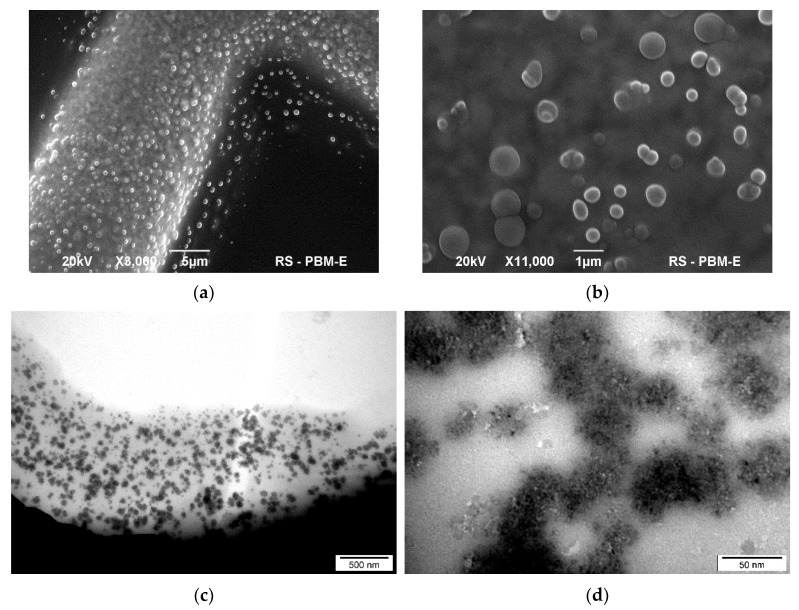
Morphology of CND/PEI-1.67: SEM (**a**,**b**) and TEM (**c**,**d**) images.

The addition of PEI10 to CNDs(aq) caused the formation of clusters of spherical shape and dimensions of approx. 0.5–2 µm, as presented in Figure 2a,b. However, similar structures were not observed in the pure PEI solution (i.e., without nanodots) of the same concentration. Hence, it can be concluded that emerging clusters are the result of interactions between carbon nanodots and PEI.

The presence of agglomerates in the CND/PEI-1.67 was further investigated by TEM (Figure 2c,d). The observed clusters are heterogeneous in terms of shape and size. As can be seen in the close-up in Figure 2d, these clusters consist of a carbon core and a polymer shell (greyish halo around carbon dots). The average size of the agglomerate was about 50 nm. Unfortunately, after adding PEI, even at low concentrations, it was difficult to obtain clear images of high quality.

It should be noted that other samples (i.e., CND/PEI-0.014 and CND/PEI-0.33) had similar morphology to that of CND/PEI-1.67.

**Figure 3:**
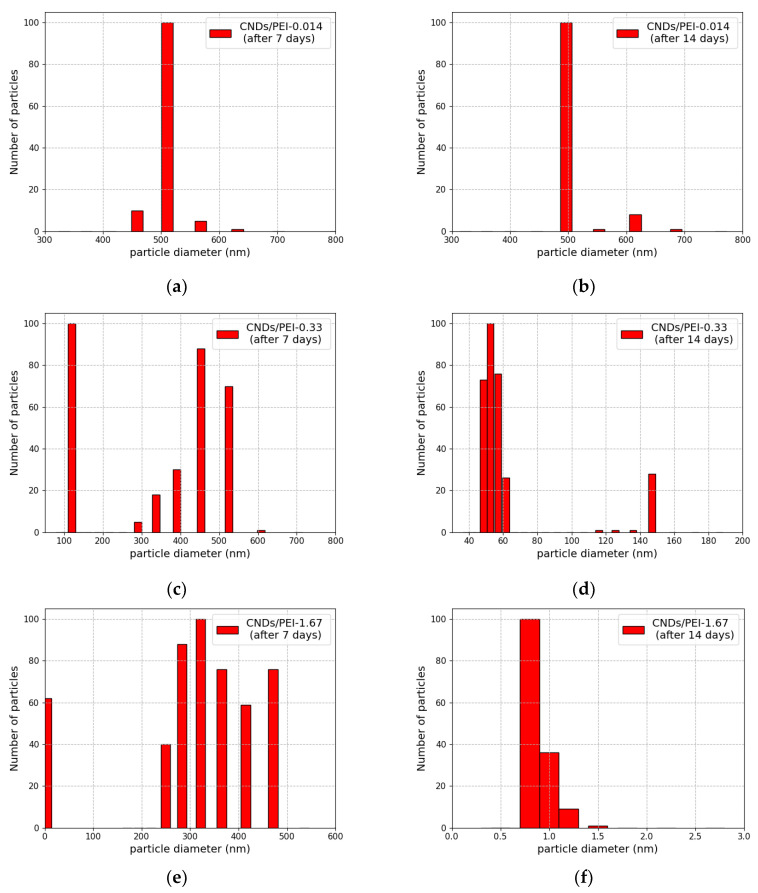
DLS size distribution of samples CND/PEI-0.014, CND/PEI-0.33, and CND/PEI-1.67 measured 7 (**a**,**c**,**e**) and 14 days (**b**,**d**,**f**) after synthesis, respectively.

The agglomeration of CND/PEI systems was also confirmed by means of dynamic light scattering (Figure 3). In comparison to CNDs(aq) (Figure 1b), all samples exhibited an increase in their dimensions after the addition of polymer to the carbon-dot colloid. The agglomeration of CND/PEI systems can be attributed to electrostatic interactions between cationic PEI and anionic CNDs, leading to the compensation of net surface charge [22,29].

It should be noted that samples CND/PEI-0.33 and CND/PEI-1.67 did not display noticeable aggregation leading to sedimentation. However, the CND/PEI-0.014 sample precipitated four days after mixing. It can be deduced that, in this case, the dosage of the polymer was insufficient to prevent the bridging or mosaic flocculation process during adsorption [31]. Therefore, it is not surprising that the size distribution of the CND/PEI-0.014 sample consisted of only large particles (500 nm), which were stable and did not increase in size between the seventh and fourteenth day after mixing (Figure 3a,b).

A comparison between CND/PEI-0.33 and CND/PEI-1.67 samples seven days after mixing (Figure 3c,e) clearly shows that both samples displayed multimodal size distribution. However, in both cases, the size of clusters diminished over time. In the case of CND/PEI-0.33 seven days after mixing (Figure 3c), both small (100 nm) and large (300–600 nm) aggregates were observed, whereas a week after (Figure 3d), there were no large clusters anymore. Similarly, in the case of CND/PEI-1.67, a decline in large fractions was observed. This can be attributed to the rearrangement and reconfiguration of the clusters. Despite the net positive charge of the formed clusters, steric repulsion was too weak to prevent van der Waals interactions between aggregates and their further reconfiguration [31].

**Figure 4:**
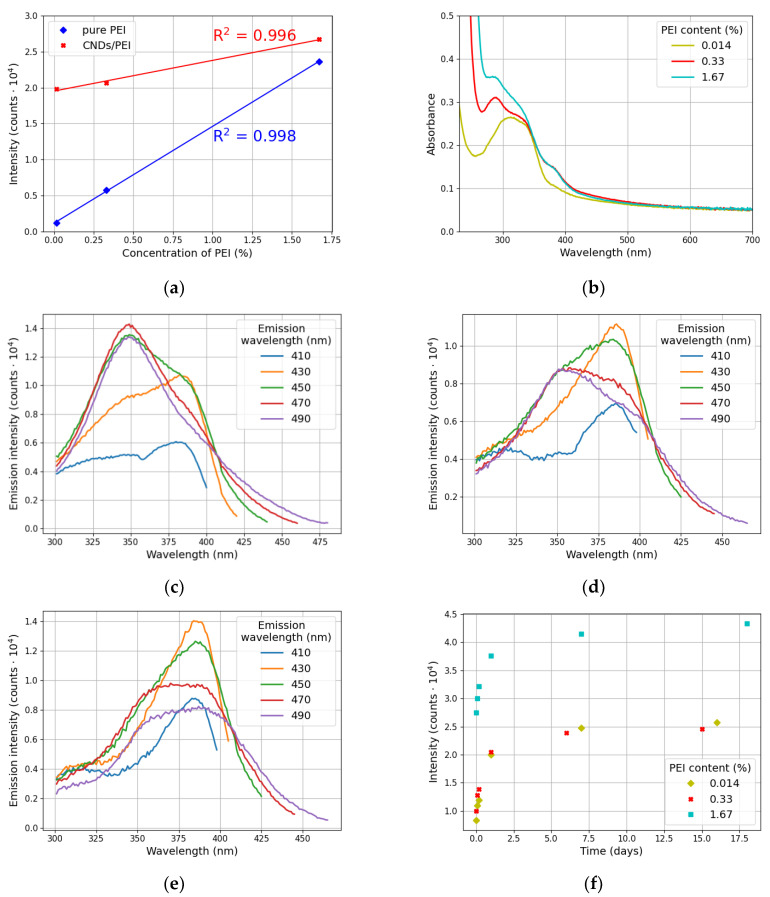
Optical properties of CND/PEI systems: (**a**) concentration-dependent emission of CND/PEI compared to pure PEI when excited at 350 nm (emission intensities were gathered for maximum emission wavelengths for pristine PEI and CND/PEI mixtures, i.e., 430 and 450 nm, respectively); (**b**) absorption spectra; (**c**–**e**) excitation spectra for CND/PEI-0.014, CNDs/PEI-0.33, CNDs/PEI-1.67, respectively; (**f**) temporal evolution of emission intensities. Absorption and emission spectra shown in Figures (**b**–**e**) were recorded 1 day after mixing the solution.

Figure 4 illustrates the optical properties of the CND/PEI systems. As shown in Figure 4a, both pure PEI and CND/PEI systems exhibited a linear dependence between the emission intensity and the concentration of the polymer in the solution. Therefore, it can be concluded that similar to pure PEI, CND/PEI systems possess an aggregation-induced emission (AIE) feature [5,24,28]. The authors are aware that the fit includes only three measurement points in both cases. Nevertheless, the high $R^{2}$ values show that the relationship between concentration and intensity can be considered linear in both pure PEI and CND/PEI mixtures.

However, as observed from Figure 4a, the linear behavior of pure polymer differs from that of CND/PEI mixtures; the emission intensity increase is more rapid in the PEI solution. This can suggest that in the presence of carbon nanodots, the process of polymer chain aggregation is slower.

Interestingly, when comparing the emission intensity values of pure polymer and CND/PEI mixtures, it can be observed that the presence of carbon nanodots in the mixture influences emission significantly for low PEI contents only (Figure 4a). It should be noted that the concentration of carbon nanodots in all CND/PEI systems was constant. Hence, higher PEI content in the solution inhibits the aggregation ratio of the system, mainly due to the steric hindrance effect [5].

Figure 4b shows the characteristic absorption bands for CND/PEI systems. CND/PEI-0.33 and CNDs/PEI-1.67 exhibited a pronounced band located at 280 nm and shoulder peaks at 340 and 380 nm. The former can be attributed to the $\pi -\pi^{*}$ the transition of the C=C skeleton [25], whereas the two latter peaks are associated with $n-\pi^{*}$ transitions [22]. CND/PEI-0.014 displayed an individual band located at 325 nm.

CND/PEI systems exerted excitation-dependent emission, as illustrated by Figure 4c–e. With an increase in the polymer content in the mixture, the maximum emission wavelength underwent a redshift (from 470 nm for CND/PEI-0.014 to 430 nm in other samples).

It should be noted that the absorbance and emission spectra presented in Figure 4b–e are representative examples of the optical behavior of the samples throughout the considered time period of 18 days after solution mixing. Indeed, no significant changes in the position of the absorbance and emission peaks were recorded during this time. However, the emission intensity of the samples increased over time.

Therefore, Figure 4f illustrates the temporal evolution of the emission intensity for all CND/PEI systems. For all polymer contents, a non-linear dependency was observed, i.e., a rapid surge in intensity values within the first day after solution mixing and emission saturation over a long time range. It should be noted that the size variations of the clusters recorded between the 7th and 14th day (Figure 3) correspond to the plateau phase of emission evolution (Figure 4f). Hence, it is concluded that the processes occurring within the first day are critical for establishing the luminescence properties of CND/PEI systems.

A gradual decrease in Stokes shift for pristine carbon nanodots and CND/PEI systems (Table 2) was observed with the increase in the polymer content in the mixture. This indicates that the amount of oscillations and vibrations is reduced by introducing polymer into the suspension [32,33]. Hence, the aggregation-induced emission of CND/PEI systems is confirmed, as this mechanism is ascribed to the reduction in vibrational motions and, subsequently, the number of non-radiative transitions [26,27].

### 3.3. Adsorption Kinetic Study

In order to provide further insight into the temporal evolution of emission intensity and, hence, the relation between the aggregation and emission of the formed CND/PEI clusters, kinetic studies of the adsorption process were conducted. Usually, the adsorption process is described using pseudo-first- and pseudo-second-order models, which can be presented in linear forms as follows:

(1) $$ln\left(q_{e}-q_{t}\right)=ln\left(q_{e}\right)-k_{1}t$$

(2) $$\frac{t}{q_{t}}=\frac{1}{k_{2}q_{e}^{2}}+\frac{t}{q_{e}}$$

where $k_{1}\;\left(\min^{-1}\right)$ is the first-order rate constant of adsorption; $q_{e}$ and $q_{t}$ represent the amounts of dyes adsorbed $\left({\mathrm{mg}\;g}^{-1}\right)$ at equilibrium and at time t (min); and $k_{2}\;\left({g\;\mathrm{mg}}^{-1}\min^{-1}\right)$ stands for equilibrium rate constant of pseudo-second-order [34,35,36].

In our experiments, it was not possible to collect data on the amounts of polymer adsorbed. Hence, it was assumed that the emission intensity value of CND/PEI systems can serve as an indirect measure of polymer molecules attached to carbon dots’ surface. This assumption is based on previously mentioned observations, concerning the substantial intensification of the emission of CND/PEI mixtures, when compared either to pristine CNDs(aq) or PEI solutions (as shown in Figure 4a). Therefore, $q_{e}$ and $q_{t}$ were replaced in Equations (1) and (2) by $I_{pol}$ and $I_{mix}$, representing the emission intensity values of pristine polymer and CND/PEI mixtures, respectively.

**Figure 5:**
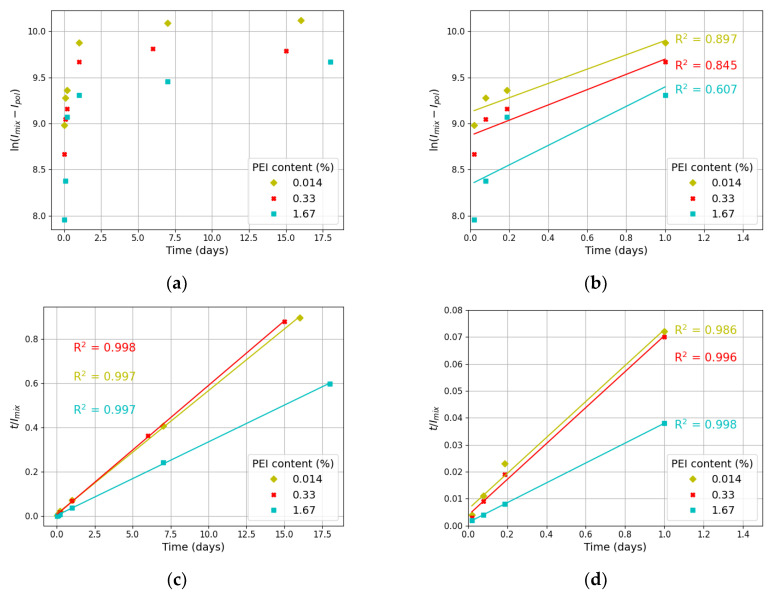
Temporal evolution of fluorescence intensity: pseudo-first-order model within 18 days (**a**) and 1 day (**b**) and pseudo-second-order model within 18 days (**c**) and 1 day (**d**) after obtaining CND/PEI solutions. For each model, $R^{2}$ correlation coefficients are included with font colors corresponding to the experimental points for each sample.

Figure 5a,b show the pseudo-first-order model, whereas Figure 5c,d illustrate the pseudo-second-order kinetic model. The $R^{2}$ correlation coefficient was computed for each model.

Firstly, it should be noted that all samples, despite the differences in their polymer content, display a similar degree of fit to both models considered. Therefore, it can be concluded that the polymer concentration in the samples is irrelevant to aggregation behavior. Thus, it can be stated that there is one clustering mechanism for all samples.

The results presented in Figure 5a,b show that experimental data deviate from linearity in both the long (measurements taken between the 1st and 18th day) and short (1st-day measurements) time range after solution mixing. This demonstrates that emission uptake (and, hence, the adsorption of polymer onto CNDs’ surface) is not governed by first-order kinetics.

By contrast, experimental data show good agreement with the pseudo-second-order equation (Figure 5c,d). Therefore, it can be concluded that the obtained results fundamentally fit the pseudo-second-order kinetics due to the high fitting quality of the data. However, as pointed out by [37], there is no reasonable explanation for this fact since the pseudo-second-order model (as well as the pseudo-first-order model) is strictly empirical, and there is no physical justification to obtain values of kinetic constant. The successive fitting of the experimental data with the pseudo-second-order model is considered to be an artificial one and is attributed to the method of data treatment [37]. Thus, despite the high fitting quality represented by high $R^{2}$ values, the pseudo-second-order model does not provide information about the aggregation mechanism of the samples.

Also, there are presumptions that adsorption kinetics is governed by the diffusion process [37]. Therefore, experimental data were analyzed using an intraparticle diffusion model in the following form:

(3) $$q_{t}=k_{i}t^{1/2}+C$$

where $k_{i}\;\left({\mathrm{mol}\;g}^{-1}\min^{-1/2}\right)$ is the intraparticle diffusion rate constant, and $C\;\left({\mathrm{mol}\;g}^{-1}\right)$ is a constant value providing information concerning the thickness of the boundary layer [34,35,36].

**Figure 6:**
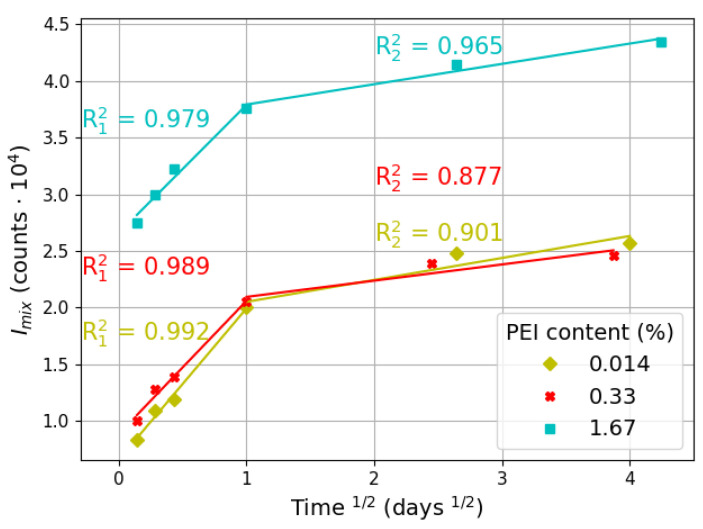
Temporal evolution of fluorescence intensity: intraparticle diffusion model. $R^{2}$ correlation coefficients for each diffusion step are included, with font colors corresponding to experimental points for each sample.

Figure 6 shows a multilinear plot of the intraparticle diffusion model of polymer adsorption on the CNDs. Again, the $q_{t}$ value was replaced by $I_{mix}$ in Equation (3).

Indeed, as shown in Figure 6, experimental data show a very good agreement with the intraparticle diffusion model within the entire investigated time range. Hence, it can be stated that the uptake of emission is controlled by the diffusion process. Also, this indicates that the aggregation mechanism of the clusters is governed by diffusion. It should be emphasized that the high fitting quality of the data (high $R^{2}$ values) to the intraparticle diffusion model is a direct proof of the validity of the abovementioned assumption that emission uptake can serve as an indirect measure of polymer molecules’ attachment to carbon dots’ surface. Moreover, all samples display a similar degree of fit (similar $R^{2}$ values) to the intraparticle diffusion model despite the differences in their polymer content. Therefore, it can be concluded that the clustering mechanism is uniform for all samples.

It should be noted, however, that the intraparticle diffusion process is gradual, as indicated by multilinear plots with different slopes $\left(k_{i}\right)$ in each of the lines present (Figure 6, Table 3). Therefore, it is suggested that at least two parameters influence emission uptake (and sorption process) [36]. Each plot line represents a distinct step of the diffusion process. As observed in Figure 6, the first stage of diffusion is rapid as emission intensity values increase sharply. The second step of the process is slower since emission intensities do not change significantly. This gradual behavior is also reflected in the calculated diffusion rates ratio presented in Table 3; it is clearly noticeable that the second stage of diffusion is around seven times slower than the initial one.

Hence, it can be concluded that the first rapid step of the diffusion process is primarily responsible for establishing the optical properties of the system. This finding is consistent with the one inferred from Figure 3 and Figure 4.

As mentioned earlier in the paper, PEI is a polycationic polymer [28], whereas CNDs are negatively charged. Hence, initially after mixing, the driving force for adsorption is electrostatic attraction between oppositely charged particles/molecules [31]. Therefore, the first diffusion step can be associated with charge neutralization, when charged PEI adsorbs on the surface of CNDs [31,38]. After charge compensation, the first stage of mixing is completed and the emission property of the system is roughly established (Figure 6).

However, since newly established molecules possess a net positive charge, they can still interact with each other, which may lead to further reconfiguration of the system. Nevertheless, as mentioned, these interactions do not influence emissions.

Also, the gradual behavior of emission uptake can be explained based on the Stokes–Einstein formula, presented in Equation (4):

(4) $$D=\frac{kT}{3\pi \eta d}$$

where D is the diffusion coefficient, k is the Boltzmann constant, T is the absolute temperature, η is the dynamic viscosity, and d is the particle diameter [39].

Table 4 summarizes the calculated diffusion coefficients using Equation (4) for exemplary sizes of emerging particles in CND/PEI systems. It is assumed that due to very low concentrations of polymer in the solution, the viscosity of water (0.89 cPa) can be adopted as solution viscosity in calculations. Also, the calculations were performed at a constant temperature of T=300 K.

Moreover, it is possible to associate the calculated diffusion coefficients with particle displacement with the following correlation of one-dimensional random walk in 3D space (5):

(5) $$x=\sqrt{2Dt}$$

where x is a particle displacement, D stands for the diffusion coefficient, and t represents time [40]. The calculated displacement values for t=24 h are presented in Table 4.

Since it is assumed that during the mixing of the CND/PEI solution, both viscosity and temperature are constant, the diffusion coefficient is inversely proportional to the particle diameter. Indeed, as can be observed from Table 4, the diffusivity of the particles decreases with the increase in the particle diameter. Therefore, the initial step of rapid diffusion can be attributed to the presence of small particles (nanodots) in the solution, and, hence, their high mobility. Due to the adsorption of PEI molecules, particles gradually increase in size and form large aggregates. Thus, diffusion slows down with clusters expanding in size [39]. The lower mobility of large aggregates translates into reduced particle displacements.

## 4. Conclusions

CNDs produced in pure water by the ablation of graphite with a nanosecond laser pulse exhibited only weak photoluminescence. A small addition of PEI to the aqueous suspension of CNDs caused a significant increase in emissions. Then, the aggregation-induced emission (AIE) mechanism of CND/PEI systems was revealed. The division of the synthesis into two distinct stages provided better control over the entire process. Thus, the first step was the production of pristine aqueous CNDs by laser ablation and centrifugation. Conducting ablation in water and limiting the amount of polymer used afterward prevented the formation of fluorophores. Hence, the first step enabled obtaining homogeneous and stable CND colloids with specific morphology, size, and optical properties, which further served as a batch for functionalization with PEI at the second stage. Therefore, this process facilitated the subsequent determination of the properties of CND/PEI systems. The results showed that CNDs were highly optically sensitive and responsive even to trace amounts of PEI in the solution (~0.014% *v*/*v*). Thus, a significant enhancement in the initial weak blue emission of CNDs and PEI taken separately was achieved. The morphology and size measurements indicated that particle aggregation occurred in the presence of a polymer. The AIE mechanism was further confirmed by the calculated Stokes shift values, which decreased with the increasing polymer content. Hence, it follows that due to molecule clustering, the number of non-radiative transitions was reduced, resulting in the enhancement of the emission intensities of CND/PEI systems. Moreover, emission properties were found to be determined within the first day after mixing, with no further effect of cluster reconfiguration on luminescence. Indeed, the presented analysis of adsorption kinetics confirmed that the AIE of CND/PEI systems is governed by a gradual intraparticle diffusion process. Moreover, it was shown that the initial stage of mixing is primarily responsible for establishing the optical properties of the system. Owing to their high optical sensitivity and aggregation in the presence of trace amounts of synthetic polymer, carbon nanodots can be perceived as a promising material for optical imaging and tracking in the field of wastewater treatment.

## Figures and Tables

**Table 1:** Description of samples.

Sample Name	Mixture Composition	Concentration of PEI in the Mixture (% *v*/*v*)
CNDs(aq)	-	0
CND/PEI-0.014	CNDs(aq) + PEI0.25	0.014
CND/PEI-0.33	CNDs(aq) + PEI2	0.33
CND/PEI-1.67	CNDs(aq) + PEI10	1.67

**Table 2:** Stokes shift for pristine CNDs and CND/PEI mixtures.

Sample	Maximum Band Position (nm)		Stokes Shift (nm) [31] $\lambda_{em}^{max}-\lambda_{a}^{max}$
	Absorbance $\lambda_{a}^{max}$	Emission $\lambda_{em}^{max}$	
CNDs(aq)	260	430	170
CND/PEI-0.014	325	470	145
CND/PEI-0.33	340	430	90
CND/PEI-1.67	340	430	90

**Table 3:** Calculated diffusion rate constants for each diffusion stage.

PEI Content in the Sample (%)	Calculated Diffusion Rate Constant $k_{i}(\times 10^{4}\frac{intensity}{{days}^{1/2}})$		Diffusion Rate Ratio $\frac{k_{i}^{1}}{k_{i}^{2}}$
	First Stage $k_{i}^{1}$	Second Stage $k_{i}^{2}$	
0.014	1.33	0.19	7.00
0.33	1.17	0.14	8.36
1.67	1.13	0.18	6.28

**Table 4:** Diffusion characteristics of particles in CND/PEI systems.

Particle Size (nm)	Diffusion Coefficient $D\;\left(\times 10^{4}\frac{nm^{2}}{s}\right)$	Particle Displacement x (mm) within 24 h
5	9812	4.12
10	4906	2.91
30	1635	1.68
50	981	1.30
100	490	0.92
200	245	0.65

## Data Availability

All data that support the findings of this study are included within the article.
